# The predictive role of impulsivity and perceived social support in psychiatric symptoms of women with methamphetamine use disorder

**DOI:** 10.3389/fpsyt.2023.1116650

**Published:** 2023-04-17

**Authors:** Shuzhe Wang, Jing Li, Yibo Li, Yuwei Xia, Yu Gong, Fuqiang Mao

**Affiliations:** ^1^Department of Psychiatry and Psychology, College of Basic Medical Sciences, Tianjin Medical University, Tianjin, China; ^2^The Second Hospital of Tianjin Medical University, Tianjin, China; ^3^Tianjin Anding Hospital, Tianjin, China

**Keywords:** methamphetamine use disorder, psychiatric symptoms, impulsivity, perceived social support, moderating effect

## Abstract

**Background:**

Currently, few studies have examined the mental states of Women methamphetamine patients, and the influence of impulsivity and perceived social support on substance misuse-induced mental disorders is unclear. We want to examine the mental state of women with methamphetamine use disorder and compare it to the Chinese norm value of healthy women. Investigate the connection between impulsivity, perceived social support and mental state of women with methamphetamine use disorder.

**Method:**

Two hundred thirty women subjects with a history of methamphetamine usage were recruited. The Chinese version of the SCL-90-R, (SCL-90) was used to evaluate psychological health problems, while the Multidimensional Scale of Perceived Social Support (MSPSS) and Barratt Impulsiveness Seale-11 (BIS-11) were utilized to evaluate perceived social support and impulsivity, respectively. The *t*-test, Pearson correlation analysis, multivariable linear regression, stepwise regression models, moderating effect analysis were used to analyze the statistics.

**Results:**

There was a noticeable difference between the Chinese norm and all participants’ SCL-90 ratings, especially for Somatization (*t* = 24.34, *p* < 0.001), Anxiety (*t* = 22.23, *p* < 0.001), Phobic anxiety (*t* = 26.47, *p* < 0.001), and Psychoticism (*t* = 24.27, *p* < 0.001). In addition, perceived social support levels and impulsivity levels are independently predictive of SCL-90 scores. Lastly, the impact of Impulsivity on SCL-90 can be modulated by perceived social support.

**Conclusion:**

According to this study, women with methamphetamine use disorder have worse mental health conditions compared to healthy subjects. Furthermore, certain psychological symptoms associated with methamphetamine use in women can be aggravated by impulsivity, while perceived social support acts as a protective factor for methamphetamine-related psychiatric symptoms. Specifically, perceived social support weakens the impact of impulsivity on psychiatric symptoms in women with methamphetamine use disorder.

## 1. Introduction

Methamphetamine (Meth) is a highly addictive stimulant (1). In recent years, there has been an increase in methamphetamine use disorder (MA) worldwide. Moreover, Meth has been the most often used and reported amphetamine in developing nations, particularly in East and Southeast Asia (2, 3). In 2020, China had 1.03 million registered drug users of amphetamine. Of these, 57.2% admitted using synthetic narcotics, primarily methamphetamine (2).

Methamphetamine intoxication affects nearly all systems of the body, causing certain pathological symptoms, including tremors, chest discomfort, nausea, vomiting, hypothermia or hyperthermia, and increased heart rate (4–6). Some methamphetamine intoxication effects can even cause death (7). In psychiatric respect, MA is associated with a lot of Psychopathological harms, known as methamphetamine (MA)-related psychiatric symptoms (MAP) (6, 8). Meth patients with MAP are more likely to have cognitive impairments (9), poor visual attention, and poor mental flexibility (10, 11). Studies have shown that in a quarter of cases, Meth patients experience psychiatric symptoms severe enough to require lifetime hospitalization (6). Notably, compared with men, women with methamphetamine use disorder experience more neuropsychiatric symptoms (12). Some studies have focused on the risk factors of MAP, revealing some potential factors responsible for MAP. The present study focused on two potential risk factors of MAP, including impulsivity and perceived social support (PSS).

Through numerous psychological questionnaire surveys and biological experiments, previous psychological research has shown that impulsivity is positively associated with several personality disorders, such as borderline personality disorder and antisocial personality disorder, as well as some clinical psychiatric symptoms, such as depression and anxiety (13–15). Regarding substance use disorder, several studies have investigated the association between impulsivity and substance misuse. For instance, using questionnaires, including the Eysenck impulsivity questionnaire, Impulsivity inventory, and Barratt impulsiveness scale, Allen found that patients with a history of Past substance dependence diagnoses may have higher impulsivity than the healthy controls (16). Studies have shown that Meth patients report more impulsivity in the abstaining period of Meth use, especially when the abstaining time is short (17). In a literature review by Brady (18), the prior studies indicated that patients with substance abuse disorders are more likely to experience impulsivity-related problems. The studies revealed that high impulsivity might be a behavioral trait of patients with substance misuse, and abstaining from the psychoactive substance in the short term may be a risk factor for impulsivity in Meth patients. Thus, we speculate that there is also an association between impulsivity and MAP. Some studies have illustrated the possibility of an association between impulsivity and MAP. For example, Using BIS-11, Su (19) found that MAP is more likely to occur in individuals with a high impulsivity than in individuals with a low impulsivity. Lapworth found that dependency on Meth is associated with higher impulsivity and the appearance of more positive psychotic symptoms (20). Moreover, MA leads to greater hostility among Meth patients (20). In addition, studies have indicated an association between MA-related impulsivity, pathological gambling (21), and violent behavior (22–24). However, whether individuals with MA are highly impulsive in remission or before they commence using the drug remains unclear (20). If so, whether this impulsivity increases MAP also remains unclear.

On the other hand, PSS refers to the extent to which an individual feels that their needs for support, information, and feedback are met (25, 26). Social support is a potent buffer against stressful life events and Pathological psychological conditions (25, 26). Studies have shown that PSS is inversely related to the development of depression (27–29), bipolar (30–33), and anxiety disorders (34–36). Moreover, a lower PSS at baseline indicates a higher symptom intensity and a poorer recovery/remission (37).

A study by Joiner suggested that social support reduces the negative impact of impulsivity on irrational acts, such as suicide and mental illnesses (38). Some researchers have indicated that social support moderates the relationship between impulsivity and depression (39), suicide (40), and parental borderline personality disorder (41). Providing social support to patients undergoing substance misuse can prevent many drug-misuse disorders (42–44). Some researchers have reported that PSS, especially in the family, can prevent individuals from drug misuse and addiction (45). In addition, recent research examining the association between PSS and MAP found an association between MAP and a lack of social support. In addition, the study found that social support could moderate the effects of genetic risk factors of MAP (8). Therefore, PSS may predict MAP and moderate the impact of impulsivity on MAP.

This study aimed to: (1) investigate the mental status of Chinese women with methamphetamine use disorder and compare it with healthy controls; (2) investigate whether PSS and impulsivity predict the occurrence of MAP in the remission period of MA and the impact of each dimension of PSS on MAP; and (3) investigate whether PSS moderates the relationship between impulsivity and MAP.

## 2. Materials and methods

### 2.1. Participants

A total of 230 women participants with MA were recruited from a drug rehabilitation center in Tianjin, China. Staff at the drug rehabilitation center organized a group test for the Meth patients, each group involving 46 patients. The inclusion criteria for participants were: (1) adults between 18 and 50 years; (2) using methamphetamine alone or in combination with alcohol or cigarettes, or in combination with both alcohol and cigarettes and diagnosed with amphetamine-type substance use disorder, according to the DSM-5; (3) a total of 3–20 months from the restricted phase(in this phase they were restricted from leaving the drug rehabilitation center); (4) with no history of brain injury or other mental illness diagnoses; (5) were not suffering from severe neurological and physical diseases; (6) subjects have both biological and sociological attributes of femininity. All subjects had received compulsory treatment in the hospital before the restricted phase. In the restricted phase, every subject was managed inpatient, without using medication or addictive substances. In addition, the patients were not allowed to leave the drug rehabilitation center alone in the restricted phase. The survey was conducted anonymously, and each subject answered the questionnaires independently. In this study, all subjects volunteered to participate and the informed consent form was signed by every participant. As compensation, we provided every patient with 2 weeks of group psychological counseling after the study had ended.

Before the test, the authenticity and confidentiality of the test were emphasized by the Institutional Ethical Review Board of Tianjin Medical University.

### 2.2. Measurement

This study assessed the mental health of Meth patients using the Chinese version of the SCL-90-R (SCL-90). The SCL-90 is a self-report symptom inventory established by Derogatis (46). with strong internal consistency and reliability. The Cronbach’s α coefficients of the SCL-90 were 0.78 to 0.92 (47–50). SCL-90 is commonly used to evaluate the psychological health of different mental illnesses (48, 51). The Chinese version of SCL-90-R includes nine dimensions and 90 items, and it takes 10–15 min to complete the questionnaire (52).

The perceived social support among methamphetamine patients was evaluated using the Multidimensional Scale of Perceived Social Support (MSPSS), with 12 items and three subscale scores for Friends, Family, and Significant Others (25, 26). Each of these groups contained four objects. To improve the diversity of answers and reduce a ceiling effect, a 7-point rating scale was devised, spanning from very strongly disagree to very strongly agree. The total-scale and respective Cronbach’s α coefficients of subscales were (0.93, 0.91, 0.87, and 0.85) (25, 26).

The impulsiveness of Meth patients was evaluated using the Barratt Impulsiveness Scale-11 (BIS-11) Chinese edition. Zhou (53) altered the Chinese version of BIS-11 to include 26 items on a scale from 1 to 4. This scale shows good reliability and validity. Here, Cronbach’s α coefficient of the total-scale and subscales were 0.7590, 0.5648, 0.6584, and 0.6872 (53). Therefore, this scale has been extensively used in China.

### 2.3. Quality control

The questionnaires were filled out anonymously, without mentioning any personal information, to avoid potential bias. Moreover, the questionnaires were completed independently by subjects and were handed out and returned by trained investigators with the permission of the drug rehabilitation centers. Lastly, we eliminated the questionnaires with poor results, such as the answers to all questions being identical or unanswered questionnaires.

### 2.4. Statistical analysis

In the beginning, the normality of each variable was checked using the Shapiro–Wilk test. Descriptive statistics were performed to report the scores of demographic variables and clinical characteristics of individuals undergoing methamphetamine use disorder. Normally distributed data were analyzed using independent sample t-tests and one-way analysis of variance (ANOVA), while non-normally distributed data were analyzed using the χ^2^ test. Differences in SCL-90 scores between respondents with various demographic features were investigated. Differences in SCL-90 average scores, across the nine dimensions, between the healthy controls (54) and women with methamphetamine use disorder were assessed using the t test. In addition, the association between SCL-90, PSS, and impulsivity was investigated using the Pearson correlation analysis. The possible variables impacting each of the nine SCL-90 features were evaluated using multivariable linear regression. Then, stepwise regression models were used to investigate the MSPSS subscales and each SCL-90 dimension. Finally, a moderating effect analysis was carried out using the PROCESS program. In this model, all variables were standardized; impulsivity was the independent variable, PSS was moderating variable, and SCL-90 was the outcome variable. Age, gender, drug use duration, abstinence duration, smoking history, drinking history, marital status, family members taking drugs, and the way of drug use were entered as covariates. All data analysis was performed using the Statistical Package for the Social Sciences (SPSS) version 25.0. *p* < 0.05 was considered statistically significant.

## 3. Results

A total of 230 questionnaires were presented to women Meth patients in the drug rehabilitation center in Tianjin, China. Of these, 228 questionnaires were enrolled for data analysis after eliminating two due to poor quality.

### 3.1. Sociodemographic characteristics

Table 1 shows the demographic and clinical information of the subjects. Among the 228 women with methamphetamine use disorder, the majority were unmarried, particularly young and middle age (≤45 years) (87.3%). Most had a drinking (63.6%) and smoking (88.2%) history. Inhaling and Smoking (94.3%) were the main routes of administration. The independent sample *t*-tests, one-way analysis of variance, and χ^2^ test revealed no significant differences in SCL-90 in all these Sociodemographic Characteristics.

**Table 1:** Sociodemographic characteristics of participants.

Characteristics	Participants	
	*n*	%
Gender		
Female	228	100.0
Marital status		
Single	88	38.6
Married/partnered	56	24.6
Divorced/widowed	84	36.8
Other	0	0
Smoking History^a^	201	88.2
Drinking History^a^	145	63.6
The main routes of administration		
Intravenous	3	1.3
Inhaling, Smoking	215	94.3
Mixture	10	4.4
Family members taking drugs^a^	32	14.0
Age (Years)		
≤30	71	31.1
31–45	128	56.2
46–60	29	12.7
Abstinence duration		
3–6 Months	16	7.0
7–12 Months	39	17.1
12–24 Months	149	65.4
>24 Months	24	10.5
Drug use duration		
≤ 5 Months	97	42.6
6–10 Months	66	28.9
11–20 Months	53	23.2
>20 Months	12	5.3

*N* = 228.

a Reflects the number and percentage of participants answering “yes” to this question.

### 3.2. Subjects’ scores in each dimension of SCL-90

The scores of subjects in nine SCL-90 factors are shown in (Table 2). All participant scores were much higher than the healthy controls for each dimension, especially in Somatization (*t* = 24.34, *p* < 0.001), Anxiety (*t* = 22.23, *p* < 0.001), Phobic anxiety (*t* = 26.47, *p* < 0.001), and Psychoticism (*t* = 24.27, *p* < 0.001).

**Table 2:** Respondents’ scores in nine dimensions of SCL-90 (*N* = 228).

Dimensions	Methamphetamine		Norm		*t*	*p*
	*M*	*SD*	*M*	*SD*		
Somatization	1.99	0.37	1.39	0.45	24.34	<0.001
Obsessive–compulsive	2.17	0.44	1.68	0.58	16.74	<0.001
Interpersonal sensitivity	1.95	0.37	1.52	0.55	17.56	<0.001
Depression	1.98	0.46	1.47	0.54	16.90	<0.001
Anxiety	1.99	0.39	1.41	0.48	22.23	<0.001
Hostility	1.85	0.42	1.50	0.57	12.62	<0.001
Phobic anxiety	1.85	0.33	1.24	0.39	26.47	<0.001
Paranoid ideation	1.83	0.40	1.40	0.48	15.93	<0.001
Psychoticism	1.85	0.31	1.34	0.43	24.27	<0.001

### 3.3. The relationship between PSS, impulsivity, and SCL-90

This study found a significant correlation between PSS and each dimension of SCL-90, except for Somatization and Hostility. In the subscales of MSPSS, “friends” only correlated with Phobic anxiety; “family” significantly correlated with each dimension of SCL-90, except for Somatization and Hostility; “others” significantly correlated with each dimension of the SCL-90, except for Somatization. There was a significant correlation between impulsivity and each dimension of SCL-90 (Table 3) illustrates the association between PSS (MSPSS), impulsivity (BIS-11), and every dimension of SCL-90 with standardized coefficients of respondents.

**Table 3:** The correlation of BIS-11, MSPSS, and respondents’ each dimension of SCL-90.

			MSPSS							
*Dimensions*	BIS-11		Family		Friends		Others		Total	
	*r*	*p*	*r*	*p*	*r*	*p*	*r*	*p*	*r*	*p*
Somatization	0.16^*^	0.028	−0.10	0.155	−0.01	0.877	−0.11	0.132	−0.08	0.250
Obsessive–compulsive	0.20^**^	0.007	−0.20^**^	0.007	−0.13	0.062	−0.19^**^	0.009	−0.19^**^	0.009
Interpersonal sensitivity	0.22^**^	0.005	−0.20^**^	0.008	−0.13	0.065	−0.20^**^	0.007	−0.19^**^	0.008
Depression	0.14^*^	0.041	−0.17^*^	0.016	−0.10	0.132	−0.21^**^	0.006	−0.18^*^	0.013
Anxiety	0.20^**^	0.008	−0.17^**^	0.017	−0.08	0.22	−0.20^**^	0.008	−0.16^*^	0.020
Hostility	0.26^**^	0.005	−0.12	0.093	−0.05	0.478	−0.15^*^	0.028	−0.12	0.095
Phobic anxiety	0.18^*^	0.014	−0.24^**^	0.006	−0.16^*^	0.02	−0.23^**^	0.006	−0.23^**^	0.005
Paranoid ideation	0.15^*^	0.028	−0.22^**^	0.006	−0.13	0.071	−0.23^**^	0.006	−0.21^**^	0.005
Psychoticism	0.18^*^	0.012	−0.18^*^	0.012	−0.08	0.250	−0.19^**^	0.009	−0.17^*^	0.020
SCL-90 (total score)	0.20^**^	0.007	−0.19^**^	0.009	−0.11	0.133	−0.21^**^	0.007	−0.18^**^	0.010

*p < 0.05; **p < 0.01. BIS-11 = Barratt Impulsiveness Seale-11; MSPSS = the Multidimensional Scale of Perceived Social Support. Each *p*-value has been adjusted by Benjamini–Hochberg Procedure. *N* = 228.

**Figure 1:**
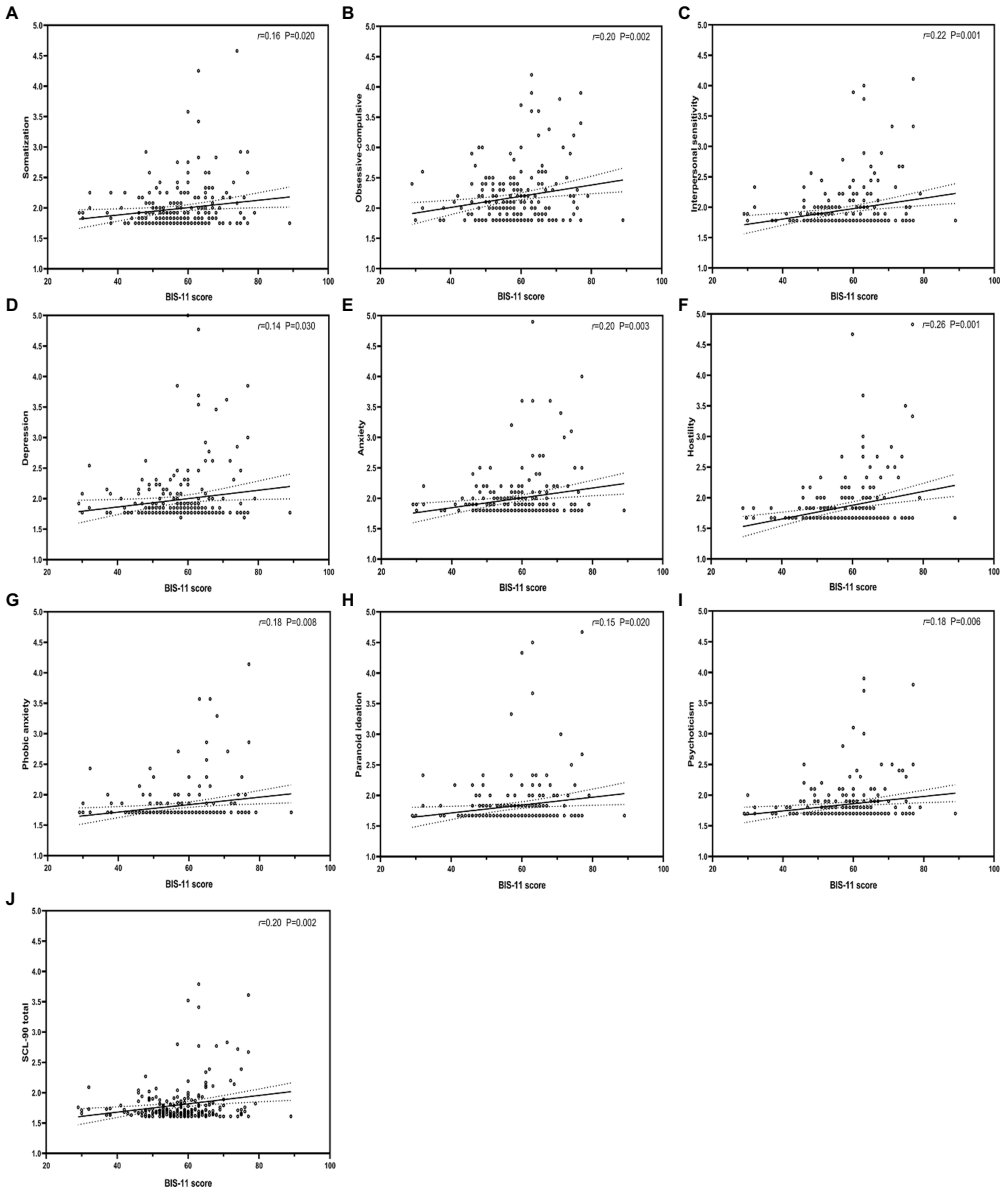
Correlation between impulsivity and each dimension of SCL-90. BIS-11, Barratt Impulsiveness Scale-11. r: Pearson correlation coefficient; p: *p*-value.

**Figure 2:**
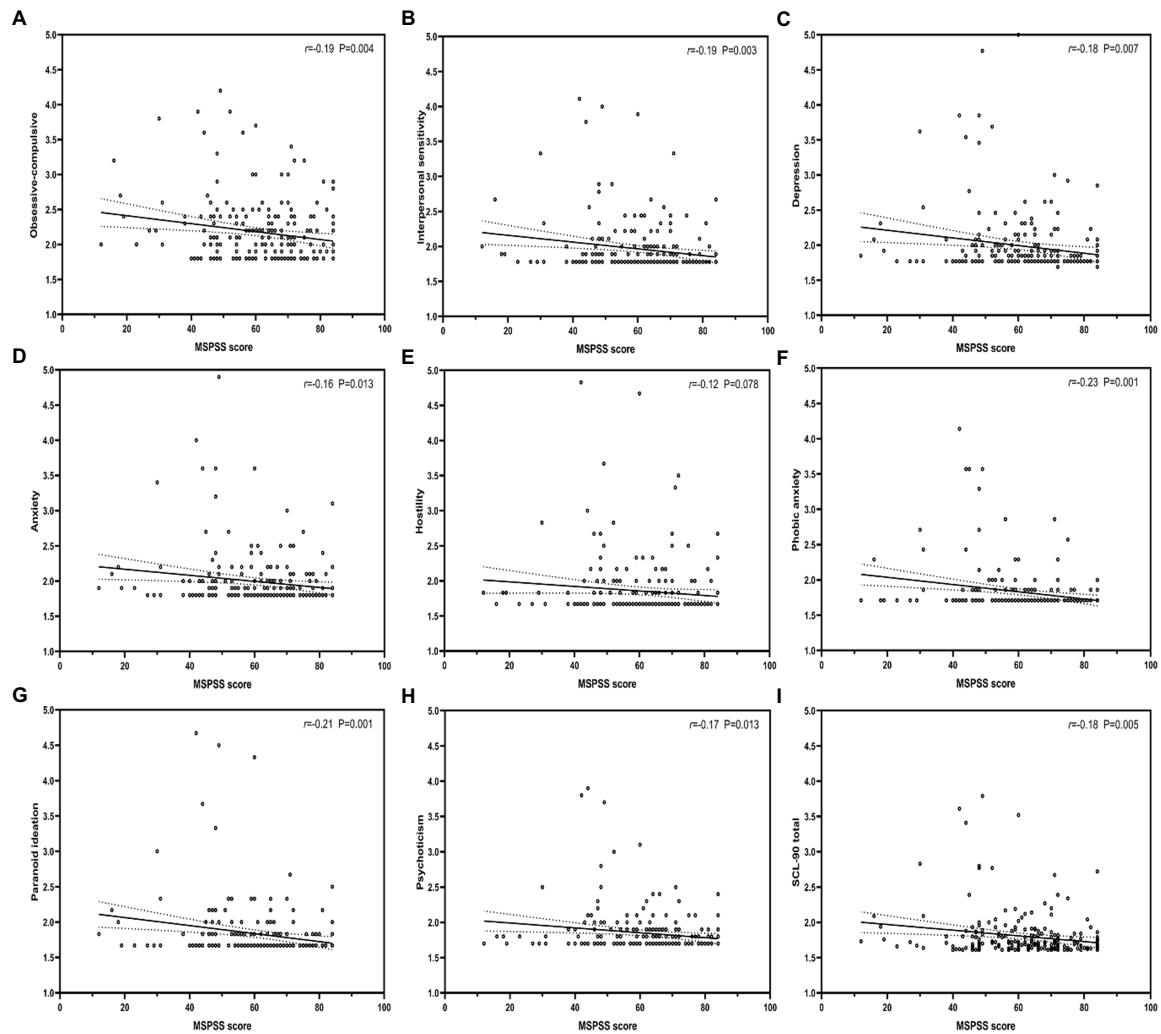
Correlation between perceived social support, and each dimension of SCL-90. MSPSS, Multidimensional Scale of Perceived Social Support. r: Pearson correlation coefficient; p: *p*-value.

The results of multivariable linear regression models of BIS-11, MSPSS, and each dimension of SCL-90 are shown in (Table 4). The results show that impulsivity positively predicted each dimension of SCL-90 (Figure 1), whereas PSS negatively predicted each dimension of SCL-90, except for Somatization (Figure 2). Further, Table 5 shows stepwise regression results of MSPSS subscales and each dimension of SCL-90. There was a significant association between “Others” and most dimensions of SCL-90, except for Somatization, Obsessive–compulsive, and Phobic anxiety. “Family” only related to Obsessive–Compulsive and Phobic anxiety, while no significant association was observed between “Friends” and SCL-90.

**Table 4:** The multivariable linear regression of BIS-11, MSPSS and each Dimension of SCL-90.

Predictors	Somatization		Obsessive–compulsive		Interpersonal sensitivity		Depression		Anxiety	
	*β*	*t*	*β*	*t*	*β*	*t*	*β*	*t*	*β*	*t*
BIS-11	0.183	2.751^**^	0.256	3.935^***^	0.279	4.333^***^	0.195	2.977^**^	0.248	3.831^***^
MSPSS (Total)	−0.120	−1.787	−0.248	−3.823^***^	−0.256	−3.975^***^	−0.222	−3.357^**^	−0.219	−3.354^**^
	*R* ^2^	*F*	*R* ^2^	*F*	*R* ^2^	*F*	*R* ^2^	*F*	*R* ^2^	*F*
Full model	0.0290^*^	4.435	0.091^***^	12.292	0.103^***^	7.838	0.059^***^	8.127	0.077^***^	10.440
Predictors	Hostility		Phobic anxiety		Paranoid ideation		Psychoticism		Total score	
	*β*	*t*	*β*	*t*	*β*	*t*	*β*	*t*	*β*	*t*
BIS-11	0.301	4.680^***^	0.240	3.710^***^	0.212	3.273^**^	0.230	3.537^***^	0.258	3.996^***^
MSPSS (Total)	−0.184	−2.833^**^	−0.283	−4.372^***^	−0.258	−3.948^***^	−0.216	−3.284^**^	−0.242	−3.717^***^
	*R* ^2^	*F*	*R* ^2^	*F*	*R* ^2^	*F*	*R* ^2^	*F*	*R* ^2^	*F*
Full model	0.091^***^	12.345	0.099^***^	13.473	0.079^***^	10.691	0.069^***^	9.367	0.088^***^	12.015

Standardized regression coefficients (β) are presented, which indicate the relative magnitude of prediction for each independent variable. The BIS-11 and the MSPSS were entered into the Multivariable linear regression models, with the significance of the explained amount of variance (*R*^2^, adjusted) for each dimension of SCL-90 assessed by using analysis of variance. *N* = 228.

^*^p < 0.05; ^**^p < 0.01; ^***^p < 0.001.

**Table 5:** The stepwise regression models of MSPSS subscales and each Dimension of SCL-90.

Predictors	Somatization		Obsessive–compulsive		Interpersonal sensitivity		Depression		Anxiety	
	*β*	*t*	*β*	*t*	*β*	*t*	*β*	*t*	*β*	*t*
MSPSS (subscales)										
Family	−0.094	−0.841	−0.200	−3.068^**^	−0.100	−0.939	−0.014	−0.128	−0.046	−0.433
Friends	0.209	1.917	0.021	0.221	0.076	0.730	0.160	1.544	0.174	1.674
Others	−0.195	−1.557	−0.089	−0.830	−0.204	−3.130^**^	−0.213	−3.275^**^	−0.195	−2.982^**^
Predictors	Hostility		Phobic anxiety		Paranoid ideation		Psychoticism		Total score	
	*β*	*t*	*β*	*t*	*β*	*t*	*β*	*t*	*β*	*t*
MSPSS (subscales)										
Family	0.010	0.093	−0.236	−3.653^***^	−0.088	−0.831	−0.076	−0.710	−0.073	−0.681
Friends	0.187	1.779	0.008	0.091	0.142	1.371	0.185	1.779	0.150	1.438
Others	**−0.154**	−2.349^*^	−0.109	−1.031	−0.233	−3.607^***^	−0.192	−2.942^**^	−0.208	−3.202^**^

Standardized regression coefficients (*β*) are presented, which indicate the relative magnitude of prediction for each independent variable. *N* = 228.

*p < 0.05; **p < 0.01; ***p < 0.001.

In moderating effect analysis, the interaction item Impulsivity × PSS coefficient was −0.0003, while the value of p was 0.0101, indicating a significant interaction between PSS and Impulsivity (*p* = 0.0101). Thus, PSS can regulate the influence of Impulsivity on SCL-90. However, the moderating effects are relatively weak. The results for combining PSS, Impulsivity, and SCL-90 are shown in Table 6.

**Table 6:** The result of moderating effects analysis of impulsivity, PSS, and SCL-90 (*N* = 228).

	Coeff	SE	*t*	*p*	LLCI	ULCI
Constant	1.799	0.1748	10.292	0	1.4543	2.1432
Impulsivity	0.0092	0.0022	4.106	0.0001	0.0048	0.0135
PSS	−0.0059	0.0014	−4.1172	0.0001	−0.0087	−0.0031
Impulsivity × PSS	−0.0003	0.0001	−2.5954	0.0101	−0.0005	−0.0001

**Figure 3:**
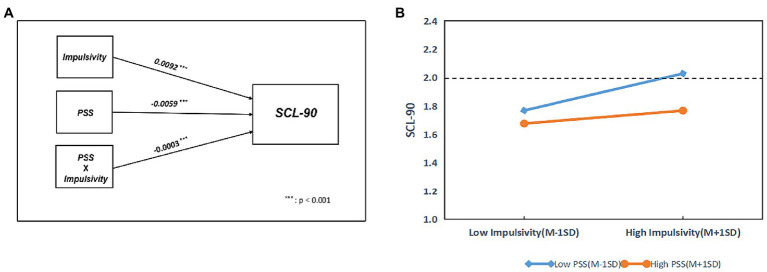
The effect of impulsivity on SCL-90 moderated by PSS. PSS, Perceived Social Support. r: Pearson correlation coefficient; p: *p*-value.

Figure 3 illustrates the conditional effect of Impulsivity on SCL-90 moderated by PSS. The impact of Impulsivity on SCL-90 was examined using a simple main effects analysis at 1 ± SD of the normalized PSS score. In each level of PSS, there was a significant association between impulsivity and SCL-90. Nevertheless, the low PSS (PSS = −1) line was steeper than the high PSS (PSS = 1). Therefore, PSS weakens the ability of Impulsivity to affect SCL-90.

## 4. Discussion

Previous studies have shown that: (1) people with substance use disorder have poorer mental status and a higher prevalence of psychiatric disorders than the norm (6, 12, 55) (2) people with high impulsivity in MA are more likely to suffer from MAP (19, 20) (3) PSS, especially family, may reduce hostility in MA patients (42–45). In addition, one study found that social support may modulate genetic effects on MAP (8).

Herein, we examined the mental health status of 228 MA women and that of healthy women using the SCL-90 scale. We then measured the impulsivity and social support levels of the subjects using the BIS-11 and MSPSS scales, respectively. In addition, we analyzed the relationship between impulsivity, social support, and various psychiatric symptoms.

Our results found that Chinese women with Meth use disorder had significantly higher SCL-90 scores than the healthy controls. In addition, impulsivity and PSS, especially “others,” were significantly associated with most SCL-90 dimensions and had a positive and negative predictive influence on SCL-90, respectively. Moreover, PSS could moderate impulsivity to lessen its effects on MAP. This study found that female methamphetamine patients had more psychiatric symptoms than healthy controls, consistent with the findings of Zhao on male methamphetamine patients (56). In addition, compared with the results of Zhao on male patients with Meth use disorder, the women patients of our study had more and worse psychiatric symptoms, consistent with earlier research findings (12). Based on the statistics of (Table 1), we inferred that even in remission, women Meth patients are more likely to experience somatization, anxiety, and pathological psychiatric symptoms than normal individuals. This may worsen the Meth use disorder since patients may choose to use Meth again to alleviate these symptoms. We also observed that during the MA remission period, impulsivity positively influenced MAP, while PSS negatively influenced MAP, consistent with previous studies (19, 20). In addition, these findings partially support the earlier speculation that PSS can predict MAP. We also found a significant correlation between impulsivity and “hostility” on the SCL-90 than other dimensions (Table 3). Studies have shown impulsivity is highly correlated with hostility in non-substance misusers (57). This may explain why people with higher impulsivity are more likely to exhibit hostile behavior and aggression when experiencing anger. Worryingly, psychoactive substances may elevate both impulsivity and hostility in patients (58). Therefore, there is a need to pay more attention and closely assess the impulsivity levels of Meth patients to prevent hostile and aggressive behaviors. In the stepwise regression, PSS of “other” showed a negative predictive effect on each dimension of the SCL-90, except for Obsessive–compulsive, Somatization, and Phobic anxiety. At the same time, PSS of “friend” showed a negative predictive effect on Obsessive–compulsive and Phobic anxiety. In addition, no association were found between “family” and the SCL-90. These findings are inconsistent with previous research (45), probably due to the diverse circumstances in which the subjects were examined. In this study, the participants were isolated and had no communication with family or friends. In previous studies, subjects were mostly unrestricted, allowing them to receive greater assistance from family and friends. Therefore, for patients in residential treatment, increasing their level of social support, especially the support of “Others”, including closed detoxification community managers and volunteers, may alleviate their MAP levels. This is of great relevance to the recovery of Meth patients. Finally, PROCESS Analyses revealed that PSS moderated the influence of impulsivity on SCL-90. Compared with the low PSS group, the SCL-90 of the high PSS group reduced to within the normal range (SCL-90 score < 2), although this moderating effect was weak. The possible reason for the moderating effect generated could be that the living environment of Meth patients in a closed community is relatively monotonous. Negative life events mainly stem from conflicts with other patients or staff, which could be transformed into impulsive emotions and affect the MAP. Social support from staff and volunteers can significantly alleviate the impact of these negative life events, thereby moderating the negative effect. Similar phenomena have been reported in some literature. For example, a study of suicide behavior in rural populations found that in rural China, a relatively closed environment, people have a single source of social support (mainly from family). When individuals face conflicts, especially when these conflicts are family-related, individuals with high levels of impulsivity may engage in suicidal behavior if the social support they receive does not meet expectations (59). Similarly, when Meth patients conflict with rehab staff or other patients, they are much more likely to have MAP if they do not have access to social support from staff or volunteers.

Notably, to the best of our knowledge, this is the first study evaluating the psychiatric conditions and the level of PSS and impulsivity among Chinese women meth patients during the remission period of Meth use. We also discovered that, unlike individuals who live in open communities, Meth patients in restricted communities are more likely to receive social support from strangers and others. The social support from this part has a more significant influence on MAP than family and friends. Lastly, we found that PSS moderates the relationship between impulsivity and MAP.

Our study found that Meth may influence the mental health of women with Meth misuse disorder. In addition, impulsivity may aggravate MAP, whereas PSS may prevent MAP and buffer the influence of impulsivity on the MAP process.

However, this study had some limitations. First, although PSS and impulsivity are substantially associated with MAP, several reasons lead to MAP. Therefore, there is a need to investigate the factors that cause MAP further exhaustively. Second, this was a cross-sectional study. Consequently, we did not investigate the psychiatric health of the subjects before to Meth use. As a result, we cannot assume that all of the psychiatric symptoms of the subjects were caused by MA. Thirdly, we were unable to identify whether there was a correlation between the impulsivity of the subjects and the acute phase of MA at the time of the study, although they had been in withdrawal for at least 3 months. Fourth, it has been reported that the BIS-11 may have some limitations: (1) the subscale scores of the BIS-11 may be related to the affective state of the subjects, such as depression and manic (60) (2) the theory that BIS-11 measures three subdomains is not empirically supported (61). Lastly, all scales in this study were self-rated, highly subjective, and may have self-report bias. In addition, the SCL-90 scale can be used to assess the severity of MAP but cannot serve as a diagnostic criterion for MAP. There is a need for future studies focusing on several factors. First, our results revealed that PSS moderates impulsivity, which implies the significance of PSS as an emotional buffer for MA patients and its possible critical function in modulating other emotion-related factors of MA patients. Second, MA patients should have a longer remission phase before impulsivity testing to reduce the effect of MA on impulsivity. Third, the SCL-90 scale employed in this study to assess the mental status is a wide symptom survey scale. Therefore, future studies investigating MAP should concentrate more on longitudinal investigations of a single symptom or Pathological psychological condition. Finally, researchers could consider using other scales, such as Multidimensional Personality Questionnaire, to evaluate the impulsivity level in broad sense.

In conclusion, our results indicate that women Meth patients have poorer mental health conditions than healthy subjects. There was a significant association between impulsivity, PSS, and MAP. In addition, impulsivity positively predicted MAP, while PSS (particularly “other”) negatively predicted MAP. Finally, PSS can moderate impulsivity, reducing its impact on mental symptoms.

## Data availability statement

The raw data supporting the conclusions of this article will be made available by the authors, without undue reservation.

## Ethics statement

The studies involving human participants were reviewed and approved by Institutional Ethical Review Board of Tianjin Medical University. The patients/participants provided their written informed consent to participate in this study.

## Author contributions

JL and YBL, were in charge of the survey and data collecting, while YWX, and YG were responsible for data analysis. SZW was responsible for the experimental design and drafting of the paper. All the writers agreed to the publishing of the final paper. All authors contributed to the article and approved the submitted version.

## Funding

The work was supported by the Predominant Education and Treatment Project of the Tianjin Drug Detoxification Bureau funded this research. This research was supported by a second-class key project of the China Judicial Administration Association for Drug Rehabilitation.

## Conflict of interest

The authors declare that the research was conducted in the absence of any commercial or financial relationships that could be construed as a potential conflict of interest.

## Publisher’s note

All claims expressed in this article are solely those of the authors and do not necessarily represent those of their affiliated organizations, or those of the publisher, the editors and the reviewers. Any product that may be evaluated in this article, or claim that may be made by its manufacturer, is not guaranteed or endorsed by the publisher.
